# Construction of an Adenovirus Vaccine Expressing the Cross-reactive Antigen AMA1 for *Neospora caninum* and *Toxoplasma gondii* and Its Immune Response in an Animal Model

**Published:** 2018

**Authors:** Lijun JIA, Huanping GUO, Mingming LIU, Yang GAO, Lei ZHANG, Hang LI, Suzhu XIE, Ningning ZHANG

**Affiliations:** 1. Dept. of Veterinary Medicine, Agricultural College of Yanbian University, Yanji, Jilin 133000, China; 2. National Research Center for Protozoan Diseases, Obihiro University of Agriculture and Veterinary Medicine, Obihiro, Hokkaido 080-8555, Japan

**Keywords:** *Neospora caninum*, *Toxoplasma gondii*, *AMA1* gene, Recombinant adenovirus, Immune response

## Abstract

**Background:**

We aimed to construct an adenovirus expressing a cross-reactive fragment of the apical membrane antigen 1 (AMA1) antigen and evaluated the concomitant immune response in BABL/c mice, allowing protection against *N. caninum* and *T. gondii* infection.

**Methods:**

The study was conducted in Agricultural College of Yanbian University, Yanji, Jilin, China In 2015–2016. Primers were designed using the AMA1 gene sequences of *N. caninum* (AB265823.1) and *T. gondii* (AF010264.1). After linearization of the plasmid ADV4-NcAMA1 and the framework plasmid pacAd5, a total of 293T cells were cotransfected and Ad5-NcAMA1 recombinant adenovirus were packed. BALB/c mice were inoculated. Simultaneously serum IgG antibody levels and IFN-γ and IL-4 cytokine levels were determined by ELISA. After immunization three times in two weeks, each group of BABL/c mice were divided into two groups, respectively given intraperitoneal inoculation by the *Neospora* tachyzoite and *Toxoplasma* tachyzoite. Then we observed the clinical symptoms and statistical survival rate of mice.

**Results:**

The level of IgG in BABL/c mice immunized with Ad5-NcAMA1 was significantly increased when compared with that of pVAX1-NcAMA1 and PBS groups (*P*<0.01). At the same time, the cytokine levels of IFN-γ and IL-4 were also higher in the Ad4-NcAMA1 group than in the control groups (*P*<0.01). Moreover, BABL/c mice immunized with Ad5-NcAMA1, pVAX1-NcAMA1, and PBS showed survival rates of 75%, 45% and 20% after *N. caninum* infection, and 45%, 10% and 0% after *T. gondii* infection, respectively.

**Conclusion:**

The adenovirus vaccineAd5-NcAMA1 could provide protective immunity against *N. caninum* and *T. gondii* infection.

## Introduction

*Neospora caninum* and *Toxoplasma gondii* are both obligate intracellular parasites and have a close relationship with one another. They are not only similar in morphology, molecular characteristics, and clinical symptoms, but also to their outcomes resulting in abortion and stillbirths amongst others. These parasites belong to the phylum apicomplexa and infect cattle, sheep and other mammals (1, 2). These two parasites can also co-infect common hosts (3). These parasitic diseases have become a worldwide problem, and affect between 2% and 20% of livestock in farms in Europe. These parasites have a significant impact on the economy and the well-being of livestock, making them an important target for preventative strategies (4, 5).

Toxoplasmosis is a zoonotic disease in humans, and although there are no reports of *N. caninum* infection in humans, *N. caninum* can infect rhesus monkeys (6). Human infection with this parasite is not impossible and should be treated as a disease with zoonotic potential.

Vaccine development for neosporosis and toxoplasmosis is still in development and there are no effective drugs for the eradication of either disease (7). Thus, current efforts focus on improving the environmental conditions of livestock, eliminating sick animals, and preventing animal contact with the definitive host feces, in an attempt could be to control neosporosis and toxoplasmosis transmission. These are not the ideal approach and that inoculation with effective vaccine is the best way to control these diseases. AMA1 of *N. caninum* and *T. gondii* is typically a single-copy transmembrane protein, expressed in both brady-zoite and tachyzoite stages (8). The antibodies raised against rNcAMA1 inhibited *N. caninum* and *T. gondii* invasion in mice, suggesting that this protein can be used as cross-reactive antigen between these two parasites (9).

In this study, we constructed an adenovirus vaccine expressing the cross-reactive AMA1 fragment and evaluated the immune response in BABL/c mice. This study would lay the foundation for the development of new vaccines against *N. caninum* and *T. gondii*.

## Materials and Methods

### Parasites and cell lines

*N. caninum* from the Yanbian strain was isolated and cultured by the Department of Veterinary Medicine, Yanbian University (10). The RH-GFP (RH strain that expresses a green fluorescence protein) strain of *T. gondii* and Vero cells were kindly provided by Dr. Xuenan Xuan Obihiro from the University of Agriculture and Veterinary Medicine, Japan in 2013. Vero and Hek293T (Gene Pharma, China) cells were cultured in Dulbecco’s Modified Eagle’s medium (DMEM, Sigma, USA) supplemented with 8% heat-inactivated fetal bovine serum (PAA, Germany) and 1% streptomycin and tetracycline (Solarbio, Beijing) at 37 °C in a 5% CO_2_ air environment.

### Amplification and cloning of N. caninum and T. gondii AMA1 gene

Primers were designed using the AMA1 gene sequences of *N. caninum* (AB265823.1) and *T. gondii* (AF010264.1) available from GenBank. P1 and P2 (Table 1) primers were designed using Oligo 6.0 software and synthesized by Invitrogen Biotechnology Co., Ltd. (Shanghai, China). The total RNA of *N. caninum* and *T. gondii* was extracted by the Trizol method according to the manufacturer’s instructions (Invitrogen Biotechnology Co., Ltd.). cDNA of the *AMA1* gene was amplified by RT-PCR using a commercial kit according to the manufacturer’s instructions (Takara, Japan). These RT-PCR products were used as a template for regular PCR of the *AMA1* genes. The PCR was carried out in a 25 μL reaction. The thermocycler conditions were as follows: denaturation for 5 min at 95 °C, followed by 30 cycles that each consisted of a denaturation step at 94 °C for 45 sec, an annealing step at 58 °C for 45 sec, and an extension at 72 °C for 1 min, and the final extension at 72 °C for 7 min.

**Table 1:** The common PCR primers sets used for amplification of AMA1

***primers***	***Oligonucleotide sequences***
P1:	5′-CCGGAATTCAGCAAAATCAAGGCGAGTA-3′
P2:	5′-CTAGGGATCCGAATCCGAAACCAGGCA-3′

The length of AMA1 fragment was 679 bp. The PCR products were resolved by electrophoresis on a 1% agarose gel. PCR products were purified using a DNA Gel Extraction Kit (TaKaRa), inserted into pMD18T Simple Vector (Takara Bio., Dalian, China), and transformed into *Escherichia coli* DH5α competent cells. The positive clones were sequenced by Shanghai Biochem Technology, China. The resulting sequences were compared with those of the reference sequences in GenBank to determine sequence identities and similarities.

### Construction of plasmid and generation of recombinant adenovirus

The *NcAMA1* gene was cut from the recombinant plasmid pMD18T-NcAMA1 digested with *Eco*R I and *Bam*H I (Takara Bio), sub-cloned into the adenovirus vector ADV4-GFP (Gene Pharma), digested with the same enzymes and termed recombinant adenovirus transfer plasmid ADV4-NcAMA1. The adenovirus transfer plasmid ADV4-NcAMA1 and backbone vector pacAd5 9.2-100 (Gene Pharma) were linearized with *Pac* I and then transfected into 293T cells to generate a recombinant adenovirus Ad5-NcAMA1 expressing the NcAMA1 protein. To identify the AMA1 sequence sub-cloned into adenovirus Ad5-NcAMA1, 5 μL adenovirus Ad5-NcAMA1 was digested with protease K at 55 °C for 2 h, 100 °C for 5 min, then centrifuged at 11000 rpm for 6 min at 4 °C to obtain the supernatant that acted as template. PCR amplification was carried out using primers P1 and P2. At the same time, Hek293T cells, cultured in 24- well plates, were infected with adenovirus Ad5-NcAMA1. The expression of Ad5-NcAMA1 in Hek293T cells was identified by western blot using anti-NcAMA1 mouse serum as the primary antibody.

Viral titer, identification, and purification The Hek293T cells were cultured in 96-well plates and infected with virus solution. The virus titer was determined using the method (BT = PFU/ml) (11). The virus Ad5-NcAMA1 was purified using a Virus Purification Kit (Biomiga, USA) according to manufacturer’s instructions.

Animal immunization experiments Sixty-eight-week-old female SPF BABL/c mice were purchased from the Laboratory Animal Center of Yanbian University, China. Animal experiments were conducted according to the guidelines for the Care and Use of Research Animals provided by Laboratory Animal Center of Yanbian University. Mice were divided into three groups and then immunized. Three groups were immunized with Ad5-NcAMA1, pVAX1-NcAMA1 and PBS three times at 2 wk intervals, respectively (n = 20/each group, Table 2).

**Table 2:** The time and dose that mice immunized with different vaccines

***Groups***	***(0 week) 1st***	***(2 wk) 2nd***	***(4 wk) 3rd***
Ad5-Nc AMA1	10^9^PFU/ml	2×10^9^PFU/ml	2×10^9^ PFU/ml
pVAX1-NcAMA1	100 μg	200 μg	200 μg
PBS	100 μL	200 μL	200 μL

### ELISA

The sera of mice were collected from blood samples of mice tail before each immunization and two weeks after the third immunization. The level of IgG in sera was tested by indirect ELISA (12). At the same time, the levels of IFN-γ and IL-4 in the sera were also evaluated by using cytokine-specific assay kits (TSZ, USA).

### Challenge infections

Two weeks after the third immunization, 20 BABL/c mice from each group were then divided into two further sub-groups. Mice in one group were injected intraperitoneally with 10^6^ *N. caninum* tachyzoites of the Yanbian strain and the other group was injected with 10^3^ *T. gondii* tachyzoites of the RH strain. The survival rate of the mice was monitored daily. The challenge of infection experiments were repeated twice.

### Statistical analysis

Any significant difference was analyzed using SPSSS 19.0 software (Chicago, IL, USA). *P*-values of <0.05 and <0.01 were considered significant and highly significant, respectively.

## Results

### Cloning and sequencing of AMA1 genes

**Fig. 1:**
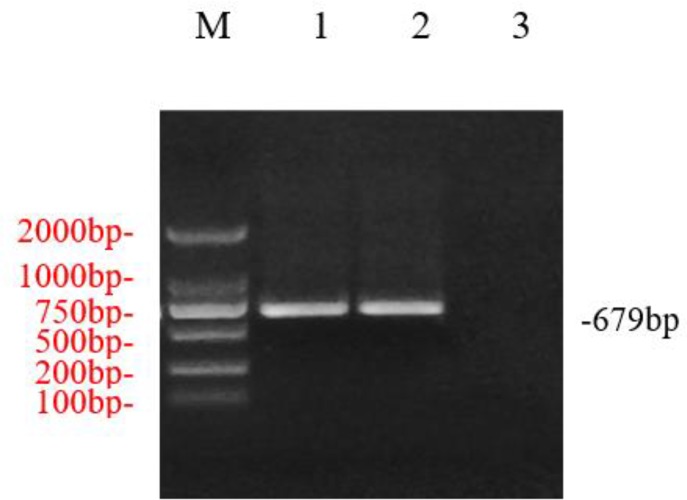
RT-PCR amplification of *AMA1* gene.M1: DL2000 DNA Marker; 1: *Neospora AMA1* gene RT-PCR amplification products; 2: *Toxoplasma gondii AMA1* gene RT-PCR amplification products; 3: Water control

Using RT-PCR, 679 bp NcAMA1 and TgAMA1 were amplified by P1 and P2 primers (Fig. 1).

PCR and restriction analyses indicated that the AMA1 genes were successfully cloned into the pMD18T vector. The 679 bp target fragments were amplified by PCR and identified from pMD18T-NcAMA1 and pMD18TTgAMA1 plasmids. The 2692 bp pMD18T and 679 bp AMA1 genes were verified by restriction analysis. The AMA1 genes of *N. caninum* and *T. gondii* had 99% nucleotide identity with published AMA1 sequences (AB265823.1) and (AF010264.1), respectively.

### Preparation of recombinant adenovirus Ad5-NcAMA1

**Fig. 2:**
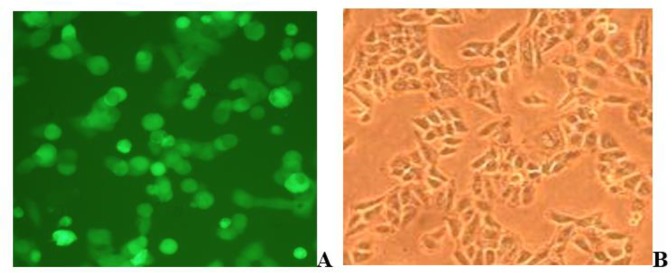
293T cells packaging Ad5-NcAMA1. A: 293T cells packaging Ad5-NcAMA1 (400 *); B: Normal 293T cells (400 *)

**Fig. 3:**
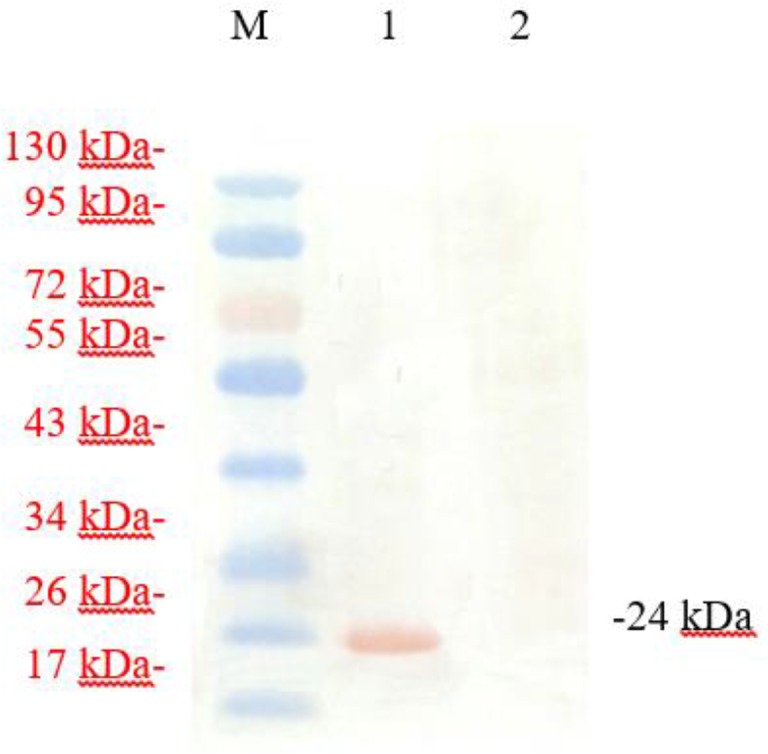
Western blotting analysis of the expression of *AMA1* gene. Lane M, Low molecular weight marker; Lane 1, expression products of Ad5-NcAMA1-infected 293 cells; Lane 2, Normal 293T cells

Using the ADV4-NcAMA1 plasmid as template, the 679 bp *AMA1* was obtained by PCR indicating that AMA1 was successfully sub-cloned into the adenovirus vector, ADV4-GFP. The adenovirus transfer plasmid ADV4-NcAMA1 and backbone vector pacAd5 9.2-100 (Gene Pharma) were linearized with *Pac* I and then co-transfected into Hek293T cells. On the eighth day after co-transfection, obvious cytopathic effects (CPE) were observed and termed recombinant adenovirus Ad5-NcAMA1 (Fig. 2). On western blotting, a specific band of approximately 24 kDa was detected in Ad5-NcAMA1-transfected 293T cells (Fig. 3).

### Viral titer, identification and purification

According to the method (BT = PFU/ml), we got a titer of 10^9^ PFU/mL, for the Ad5-NcAMA1 virus. This virus was purified using Virus Purification Kits (Biomiga, USA) and was used as vaccine for immunization in mice.

### Humoral responses induced by viral vaccine

**Fig. 4:**
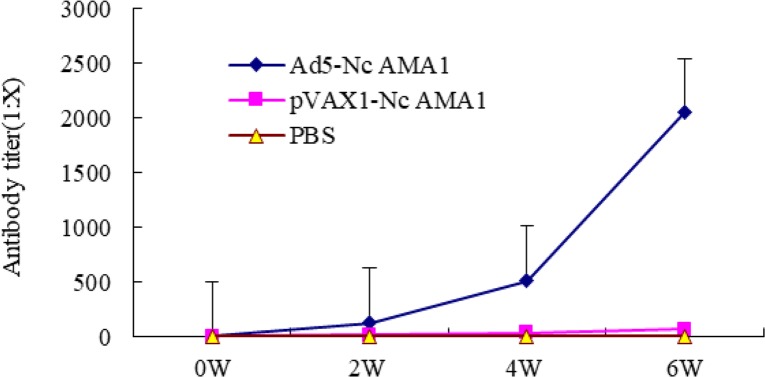
IgG levels in serum of BALB/c immunized mice in different immune groups

Two weeks after the third immunization, the level of IgG in mice immunized with Ad5-NcAMA1 was significantly higher than those immunized with pVAX1-NcAMA1 (*P*<0.01) or PBS (*P*<0.01) (Fig. 4). The recombinant adenovirus Ad5-NcAMA1 was able to induce a humoral response in the immunized mice.

### Production of IFN-γ and IL-4 in serum of mice

**Fig. 5:**
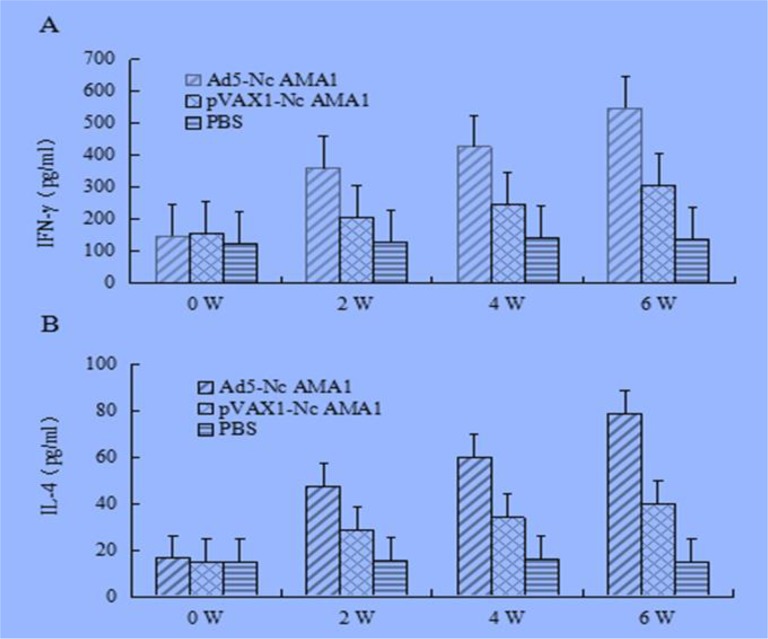
**A.** IFN-γ levels in serum of BALB/c immunized mice in different immune groups **B.** IL-4levels in serum of BALB/c immunized mice in different immune groups

The levels of IFN-γ and IL-4 in serum of the mice in this study were also evaluated using cytokine specific assay kits. The levels of IFN-γ produced by Ad5-NcAMA1 were higher than that of pVAX1-NcAMA1 (*P*<0.01) and PBS (*P*<0.01)-immunized mice (Fig. 5A) after the third immunization. Ad5-NcAMA1-immunized mice had significantly higher levers of IL-4, as compared with the mice immunized with pVAX1-NcAMA1 (*P*<0.01) and PBS (*P*<0.01) (Fig. 5B) after the third immunization.

### Protective immunity against a challenge infection with N. caninum and T. gondii following immunization

After challenge infection with *N. caninum* and *T. gondii*, no obvious clinical symptoms were observed in mice immunized withAd5-NcAMA1.

**Fig. 6:**
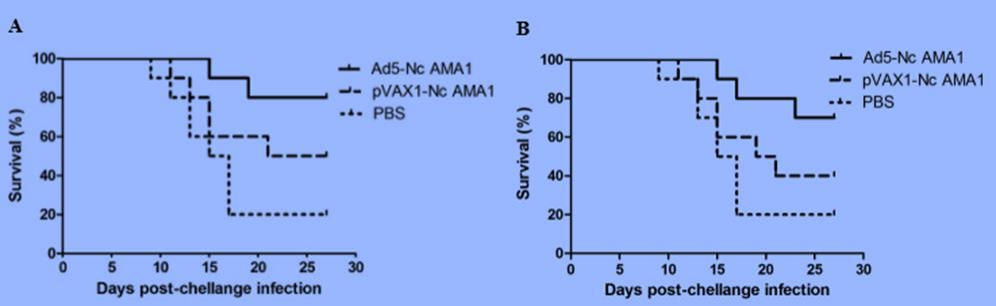
**A.** Survival curves of immunized mice in experiment 1 after *N. caninum* infection **B.** Survival curves of immunized mice in experiment 2 after *N. caninum* infection

**Fig. 7:**
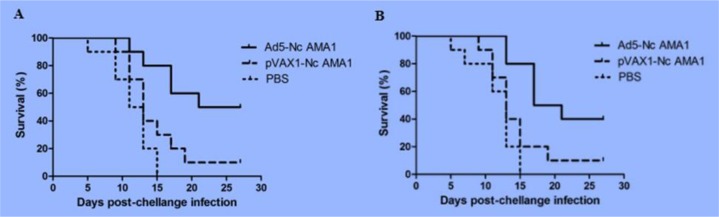
**A.** Survival curves of immunized mice in experiment 1 after *T. gondii* infection **B.** Survival curves of immunized mice in experiment 2 after *T. gondii* infection

However, mice immunized with pVAX1-NcAMA1 and PBS showed clinical signs, such as depression, hair inverted bristled, quadriplegic amongst others; these signs were more obvious in mice challenged with *T. gondii* having higher mortality rates. In two independent challenge infection experiments, BABL/c mice immunized with Ad5-NcAMA1 showed survival rates of 75% after *N. caninum* infection, while those immunized with pVAX1-NcAMA1 and PBS showed survival rates of 45% and 20% after *N. caninum* infection, respectively (Fig. 6A and Fig. 6B). The recombinant adenovirus vaccine Ad5-NcAMA1 could provide protective immunity against *N. caninum.* Moreover, BABL/c mice immunized with Ad5-NcAMA1, pVAX1-NcAMA1, and PBS showed survival rates of 45%, 10% and 0% after *T. gondii* infection, respectively. Ad5-NcAMA1 could also provide protective immunity against *T. gondii* (Fig. 7A and Fig. 7B).

## Discussion

*N. caninum* and *T. gondii* are not only similar in genetics, immunology, morphology and biological characteristics, but also cause abortion, stillbirth, and other clinical symptoms. Misdiagnosis often occurs in many cases as a result of the similarity of their clinical symptoms (13). Although the definitive hosts for *N. caninum* and *T. gondii* are different, cattle and sheep are the common intermediate hosts for both and can be co-infected (14). Therefore, it’s important to develop an effective vaccine that can inhibit both parasites.

*AMA1* genes of all parasites belonging to the phylum Apicomplexa have significant homology, and Anti-rNcAMA1 antibodies induced by rNcAMA1 in mice inhibited the infection of *N. caninum* and *T. gondii*, AMA1 was a cross-reactive antigen between *N. caninum* and *T. gondii* (15). Out of the three domains of the AMA1 protein, D I and D II play a significant role in inhibition. D II can also cause cellular immunity and humoral immunity in mice (16). Therefore, AMA1 has become a promising candidate antigen for vaccine development.

Adenovirus is a non-enveloped polyhedral virus and has been constantly developing after application in clinical trials. The genome of Ad2 and Ad5 are well described. Additionally, adenovirus has been shown to have minimal adverse effects beyond minimal symptoms and is a non-integrating virus reducing the risks of chromosomal instability in the host (17). For animals that have maternal antibodies, recombinant adenoviral vector vaccines can continue to produce antibodies and boost immunity (18). In this study, the Gene Pharma adenovirus system was used. In this system, the 5’ ITR, E1 sequence and packing signals were removed, so that the risk for viral replication within the host is reduced. Moreover, Gene Pharma adenovirus system is simple and fast for users, and only the targeted gene express a protein, and the integrated GFP allows for easy observation.

A plasmid coding for NcGRA7 as a vaccine candidate could confer partial protection against vertical transmission of *N. caninum* (19). In addition, DNA vaccine of GRA5, SAG1, and ROP2 against *T. gondii* increased level of IFN-γ in BALB/c mice (20). CpG, as an adjuvant for the NcGRA7 DNA vaccine, could significantly improve protective immunity (21). Recombinant herpes and pox virus vaccines were constructed, and the recombinant herpes virus vaccine-immunized canines could produce IgG against *N. caninum,* and showed no clinical symptoms, but also were able to resist completely canine herpes virus infection (22). Recombinant poxvirus vaccines could induce protective immunity in mice (23). Recombinant adenovirus Ad5-NcSAG1 and Ad5-NcSAG1-immunized mice were constructed and showed a positive effect on the immune response (24). Mice immunized with Ad5AMA1 and prime-boost strategy were studied and reported survival rates of 37.5% and 50% after PLK-GFP infection, respectively (25). In this study, we constructed recombinant adenovirus Ad5-NcAMA1 and evaluated its immune response in mice. The vaccine is capable of enhancing the potent humoral and cellular immune response against *N. caninum* and *T. gondii* infection.

## Conclusion

The titer of the recombinant adenovirus Ad5-NcAMA1 was 10^9^ PFU/mL. BABL/c mice immunized with Ad5-NcAMA1 showed humoral and cellular immune responses and survival rates of 75% and 45% after *N. caninum* and *T. gondii* infection, respectively.
